# Education Research: “Simulation Sharing”: Use of a Cardiac Arrest Management Course Developed for Medicine Residents Applied to Neurocritical Care Fellows

**DOI:** 10.1212/NE9.0000000000200228

**Published:** 2025-07-29

**Authors:** Daniel S. Harrison, Amjad Elmashala, Rashid A. Ahmed, Matthew F. DiFrancesco, Eliza Cricco-Lizza, Andrew Blake, Hanna Vollbrecht, Sahar F. Zafar, Matthew B. Bevers

**Affiliations:** 1 Department of Neurology, Boston Medical Center, MA;; 2 Department of Neurology, Boston University School of Medicine, MA;; 3 Department of Neurology, Mass General Brigham, Boston, MA;; 4 Department of Neurology, Harvard Medical School, Boston, MA;; 5 Department of Medicine, Brigham and Women's Hospital, Boston, MA;; 6 Department of Medicine, Harvard Medical School, Boston, MA;; 7 Department of Gastroenterology, New York Presbyterian Weill Cornell, NY;; 8 Division of Pulmonary Diseases and Critical Care Medicine, University of North Carolina at Chapel Hill, NC; and; 9 Department of Medicine, University of Chicago, IL

## Abstract

**Background and Objectives:**

Neurologists, especially neurointensivists, may be expected to lead cardiac arrest resuscitations. However, neurocritical care (NCC) fellows may face barriers to acquiring the necessary skills and knowledge needed for successful leadership in these scenarios. Whether a simulation course created for one group of learners and applied in a new context (a “shared simulation”) could facilitate acquisition of desired outcomes among neurology learners is unclear.

**Methods:**

In this prospective, pre-post educational intervention study, NCC fellows at 2 centers completed precourse knowledge and confidence assessments and reported barriers to resuscitation leadership. Fellows then led 2 simulated cases of cardiac arrest, initially developed for internal medicine residents with vignettes adapted to better reflect an NCC patient population. Postcourse knowledge and confidence assessments were administered immediately after the intervention and again one to 4 months later. Pre-, immediate post-, and delayed post-confidence and knowledge assessments were compared.

**Results:**

Thirteen NCC fellows participated in the study. Limited experience leading a resuscitation and not being preassigned the resuscitation leader role were the most highly cited barriers to leading resuscitations (n = 8/13, 61.5%). Lack of confidence and lack of knowledge were barriers for 38.5% (n = 5/13) and 22.2% (n = 2/9) of participants, respectively. Both confidence and knowledge scores improved on the immediate postassessments (5-point Likert median [IQR] 3.5 [3.1–3.9] vs 4.1 [4.0–4.7], *p* = 0.005; mean [SD] 69.8% [8.8%] vs 88.5% [7.6%], *p* = 0.004). No confidence or knowledge decay was observed between the immediate and delayed postcourse assessments (4.1 [4.0–4.7] vs 4.1 [3.2–4.4], *p* = 0.50; 91.7% [6.8%] vs 81.3% [8.0%], *p* = 0.08).

**Discussion:**

Shared simulation training improved learner confidence and knowledge in cardiac arrest resuscitation leadership and may yield similar benefits in other simulated scenarios. Low confidence, identified as a barrier to resuscitation leadership for over one-third of NCC fellows, was improved by brief simulation training.

## Introduction

Top-performing hospitals for in-hospital cardiac arrest (IHCA) generally rely on designated resuscitation teams rather than ad hoc teams.^1^ However, smaller hospitals or those with limited funding may lack the resources to train and staff such teams. This creates a challenge for neurology and neurocritical care (NCC) trainees at centers where a designated team leads resuscitation efforts who may go on to practice in hospitals where they are expected to lead ad hoc resuscitation teams. Moreover, effective resuscitation leadership is a skill that should be mastered before assuming roles where it is required, as timely execution of critical actions during a resuscitation, such as delivering an early shock in ventricular fibrillation (VF) arrest or administering early epinephrine in pulseless electrical activity (PEA) arrest, has bearing on patient outcomes.^2^

For these reasons, Accreditation Council for Graduate Medical Education (ACGME) requirements for NCC programs in particular include advanced knowledge of cardiorespiratory resuscitation.^3^ The ACGME does not require a minimum number of cardiac arrest resuscitations led as a graduation requirement for NCC fellows; however, other specialties suggest that a minimum of 45 resuscitations are required before independent practice.^4^ As IHCA is a relatively uncommon occurrence, neurology trainees likely require training in addition to chance occurrence of IHCA while on service to be adequately prepared for independent management of this problem.

Although self-reported confidence does not necessarily predict performance among neurology learners, evidence from trainees in other specialties suggests that confidence correlates with experience and training in resuscitation leadership.^5,6^ Self-efficacy, defined as a person's belief in their ability to achieve a specific outcome, positively correlates with performance and partly depends on mastery experiences.^7,8^ Neurology and NCC trainees may face limitations in developing self-efficacy and, in turn, mastery of resuscitation skills because resuscitation teams are the exclusive recipients of dedicated practice in some hospitals.

Cardiac arrest resuscitation was not identified as a common case topic in neurology residency or neurocritical care fellowship training in recent reviews, although this training has been reported in some programs.^9-11^ It is possible that a lack of case availability, previously identified as a barrier to implementing simulation in neurology residency, could also limit incorporation of cardiac arrest management training.^10^ A dedicated neurology case library was proposed and has since been created as a strategy to mitigate this barrier.^12^ However, it remains unclear that “shared simulations” (those developed by one group and implemented by another) will have the desired effects on learner outcomes.^13^

Cardiac arrest resuscitation simulation has been shown to improve learner confidence, knowledge, performance, and even patient outcomes in other contexts.^14^ Because it has been so extensively studied and understood, it serves as an excellent model for studying less well-understood phenomena in simulation, such as simulation sharing, which could be applied across disease states. As recently as 10 years ago, sharing of simulation was considered “virtually nonexistent”.^15^ Since that time, case libraries, such as MedEDPORTAL, have grown and a new neurology-specific case library has been announced.^12,16^ It is clear that cases from libraries like these are accessed.^17^ Others have investigated whether simulation sharing saves time for educators and even whether sharing of summative simulation facilitates valid learner assessment.^18,19^ Despite now many years of educators sharing simulations, it is less well established that shared formative simulations affect learner outcomes, such as knowledge and confidence, as intended.

In this study, we aimed to evaluate whether lack of confidence serves as a barrier to cardiac arrest leadership among NCC fellows, assess whether brief simulation training improves learner confidence, and determine whether a shared simulation can increase knowledge and confidence of neurology learners when applied in a new context.

## Methods

This was a prospective pre/posteducational intervention study conducted at 2 sites. Participants included junior and senior NCC fellows. All 2022 Mass General Brigham (MGB) NCC fellows were invited to participate in the course. The course was mandatory for MGB NCC fellows in 2023 and 2024. In addition, all 2024 Boston Medical Center NCC fellows were invited to participate in the course. Both sites require fellows to maintain Advanced Cardiovascular Life Support (ACLS) certification. Participation in the course was allowed even if fellows declined to participate in the study at both sites. No sample size calculation was performed, as the goal was to enroll as many fellows as possible during the study period.

### Conceptual Framework

The approach to selecting theoretical frameworks in the design of the study was driven by its individual aims. Whether lack of confidence serves as a barrier to cardiac arrest leadership among NCC fellows and whether brief simulation training improves learner confidence was guided by self-efficacy theory, a key component of social cognitive theory, as described in the introduction. Behavioral theory (which specifies that consequences or outcomes of specific actions shape learning), constructivist theory (that learners develop knowledge through experience rather than instruction), and social cognitive theory (that knowledge may be acquired by observing the experiences of others) informed the choice to study shared simulation rather than a different type of shared educational intervention in the context of cardiac arrest resuscitation training.^20^

### Study Protocol

**Figure 1:**
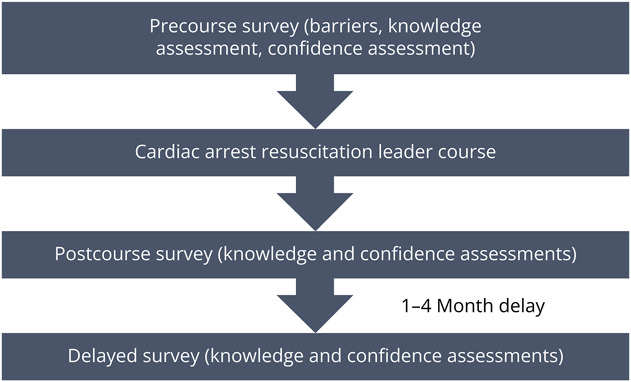
Study Protocol

The study protocol is presented in Figure 1. Participants completed a precourse survey that assessed their clinical experience, training history, and confidence. After the first year of the course, a knowledge assessment was added to the survey. Fellows then participated in 2 simulated cases of cardiac arrest: a PEA arrest due to hypoxemia and a VF arrest due to myocardial ischemia. The participants then took an immediate postcourse survey with the same knowledge and confidence assessments. Finally, 1 to 4 months after the intervention, participants completed a delayed postcourse survey which included the same knowledge and confidence assessments. We allowed for flexibility in the time window to complete the delayed postcourse survey to allow as many participants as possible to complete the follow-up assessments in the context of busy clinical schedules.

### Intervention

Each participant completed the course and participated in the study alone or with one cofellow. The course included 2 simulated scenarios of cardiac arrest, each followed by a debrief. When fellows participated in the simulation in pairs, each fellow led one case and delegated responsibilities to the second fellow, who was embedded in the case. The course was conducted at the Brigham and Women's Hospital (BWH) STRATUS Center for Medical Simulation, using high fidelity simulation resources, including a SimMan3G manikin and monitor. Each course was run by 2 members of the study team, including senior NCC fellows, senior internal medicine residents, or an NCC attending with prior cardiac arrest management training. One course faculty member acted as an embedded simulation person, playing the role of the bedside nurse, and another operated the manikin and monitor. The course was preceded by a “prebrief” to introduce the purpose of the activity, ensure participant confidentiality, plan contingencies in case of a real emergency, and establish a fiction contract (an acknowledgement that, despite the efforts of the instructors to mimic reality, there are inherent limitations to how closely real life can be simulated followed by a request that participants do their best to engage in the simulation as if it were real). Participants were instructed to respond as they would to a real patient scenario. Each simulated case was followed by a debrief, conducted by study team members using the “Debriefing with Good Judgement” approach.^6^ At least one debriefer for each session was trained through the Center for Medical Simulation (CMS) design and debriefing or STRATUS instructor course.

### Cases

Both cases were initially developed by internal medicine simulation chief residents in collaboration with an attending internist at BWH. These cases were subsequently adapted by an NCC fellow with formal training in simulation design at CMS working together with one NCC faculty member from BWH and another from Massachusetts General Hospital. Minimal changes to patient vignettes were made to better reflect the NCC patient population (e.g., a case of PEA arrest due to aspiration in the setting of mucositis was revised to a case of PEA arrest due to aspiration in the setting of status epilepticus). The learning objectives, case flow, desired learner actions, and other components of the case were not changed.

#### Case 1 Description

A 57-year-old woman with a history of epilepsy was admitted to the neurology service with focal motor status epilepticus requiring rapid uptitration of antiseizure medications. The participant is called to the room after the patient is found without a pulse. ACLS is already in process on participant arrival, at which point the participant is expected to assume leadership of the event. The participant learns that the patient had become increasingly somnolent and hypoxic throughout the shift, making hypoxemia due to aspiration in the setting of poor level of arousal the most likely etiology of the patient's PEA arrest. Return of spontaneous circulation (ROSC) is achieved after 3 rounds of cardiopulmonary resuscitation (CPR). The patient remains hypoxic and hypotensive. The case ends when the learner calls for an advanced airway and starts a vasopressor.

#### Case 2 Description

An 83-year-old man with a history of coronary artery disease complicated by ischemic cardiomyopathy presents to an outside hospital with acute onset right middle cerebral artery syndrome. He is transferred to the neurosciences ICU for consideration of endovascular therapy; however, the thrombus was too distal, and thrombectomy was not pursued. He is noted to have several runs of nonsustained ventricular tachycardia on telemetry and endorses a history of chest pain several days prior. He becomes unresponsive, and the participant is called to the room. When the participant arrives to assess, the patient is pulseless. The participant is expected to initiate ACLS and recognize that the patient is in VF arrest. ROSC is achieved after 3 rounds of CPR. The patient is comatose, and EKG review is concerning for new ischemic changes. The case ends when the learner calls a cardiology consult for consideration of left heart catheterization and initiates temperature control.

### Outcomes

The primary outcome was the change in confidence between precourse and postcourse. Secondary outcomes included change in knowledge (presurvey vs postsurvey and postsurvey vs delayed survey) and decay in confidence (postsurvey vs delayed survey). Perceived barriers to cardiac arrest leadership were also assessed on the precourse survey.

#### Confidence Assessment

A previously described assessment with validity evidence for pediatric resident confidence in cardiac arrest resuscitation skills was adapted by the study team for NCC providers (eAppendix 1–3).^9^ This instrument asked participants to rate their agreement (5-point Likert scale: 1 = strongly disagree, 5 = strongly agree) with statements regarding their confidence in executing technical skills (e.g., recognizing different cardiac arrhythmias) and nontechnical or “relational” skills (e.g., delegating tasks) during a cardiac arrest. Questions from the previously described instrument that were not relevant to adult medicine or resuscitation leadership were removed. Two questions regarding postarrest care were added based on consensus of the study team given the relevance to the target population.

#### Knowledge Assessment

A knowledge assessment was developed by the study team, which included one NCC fellow and NCC faculty members at 2 institutions (eAppendix 1–3). Participants were assessed on topics such as reversible causes of cardiac arrest, medication and defibrillator management, rhythm identification, and extracorporeal CPR. The questions and their answers were based on 2020 American Heart Association Guidelines for Cardiopulmonary Resuscitation and Emergency Cardiovascular Care.^2^

The final instruments (including knowledge and confidence assessments) were piloted and revised by consensus of the study team. The instruments were not externally reviewed.

### Data Analysis

Data were collected using REDCap and analyzed in SPSS v 28.0.0.0. Practice setting and training history data and results of assessment of barriers to code leadership are reported with descriptive statistics. Confidence assessments were compared using Wilcoxon signed-rank tests. Knowledge assessments were compared using paired *t* tests.

### Standard Protocol Approvals, Registrations, and Patient Consents

This study was approved by the MGB Institutional Review Board. Consent to participate in the research was implied by completion of the surveys.

### Data Availability

On reasonable request, the data that support the findings of this study are available from the corresponding author.

## Results

During the study period, there were 26 NCC fellows at the participating institutions, and 21 fellows enrolled in the cardiac arrest leadership course. All fellows who started the course completed the course. Two fellows were ineligible to participate in the study due to their role on the study team. Of the remaining 19 fellows who took the course, 13 participated in the study (took the precourse and postcourse assessments), 11 of whom were fellows at MGB. Details of participant training history and clinical experience are presented in Table.

**Table:** Participant Clinical Experience and Training History

Prior default resuscitation leader	23.1% (n = 3/13)
Prior simulation resuscitation leader training	66.7% (n = 6/9)
Average prior resuscitations (led)	2.0 (SD = 2.6)
Average prior resuscitations (participated, but not led)	5.8 (SD = 3.3)
Prior neurology residency training	84.6% (n = 11)
Prior neurosurgery residency training	7.7% (n = 1)
Prior internal medicine residency training	7.7% (n = 1)

Limited experience leading a resuscitation and not being preassigned the resuscitation leader role were the most highly cited barriers to leading resuscitations (n = 8/13, 61.5%). Lack of confidence and lack of insufficient knowledge were barriers for 38.5% (n = 5/13) and 22.2% (n = 2/9) of participants, respectively.

**Figure 2:**
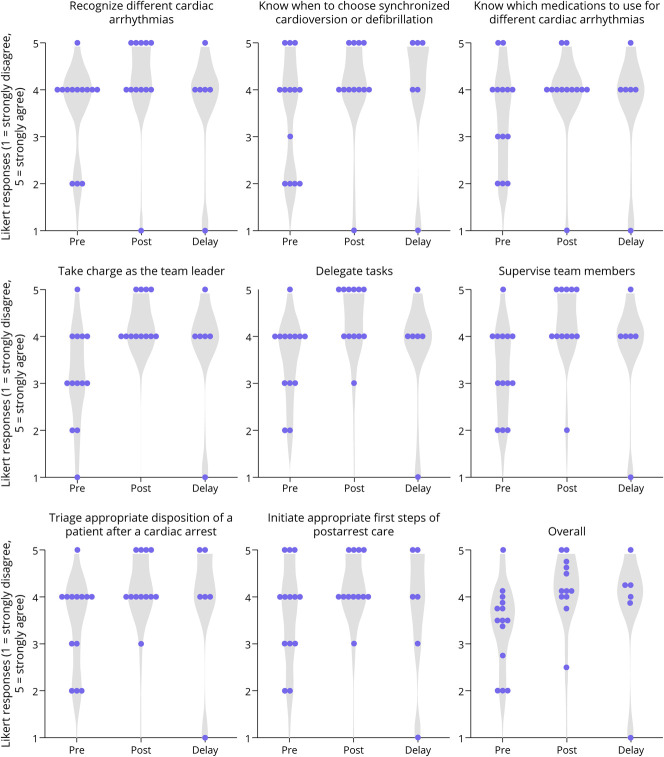
Likert Responses (5-Point Scale, 1 = Strongly Disagree, 5 = Strongly Agree) for Individual Confidence Assessment Items at Pre-, Immediate Post-, and Delayed Post-Time Points

Overall confidence in cardiac arrest management increased after the intervention (median [IQR] 3.5 [3.1–3.9] vs 4.1 [4.0–4.7], *p* = 0.005). On average, the delayed assessment occurred 69.8 days (SD 31.4 days) after the immediate postcourse assessment. There was some participant attrition, and only 6 participants completed the delayed confidence assessment. No significant decay in confidence was observed between the immediate and delayed postcourse assessments (4.1 [4.0–4.7] vs 4.1 [3.2–4.4], *p* = 0.50). Participant responses to individual confidence assessment items are presented in Figure 2.

Knowledge improved from the preassessment to the immediate postassessment (mean [SD] 70.8% [7.7%] vs 87.5% [8.9%], *p* = 0.007). Significant knowledge decay was not observed between the immediate and delayed postcourse assessments (91.7% [6.8%] vs 81.3% [8.0%], *p* = 0.08). However, a lower delayed postcourse assessment by a single participant would have resulted in statistically significant knowledge decay. Item by item knowledge assessment scores are included in the supplement (eAppendix 4).

## Discussion

Over one-third of NCC fellows cited a lack of confidence as a barrier to leading cardiac arrest resuscitation and brief simulation training improved confidence. This established simulation course, initially designed for internal medicine residents, was additionally successful in improving NCC fellow knowledge in resuscitation management.

Despite study sites requiring fellows to maintain ACLS certification, baseline scores on the knowledge and confidence assessments were low but improved after the intervention. This supports the American Heart Association recommendation that low dose, high-frequency training (rather than bolus training) should replace or supplement the current model for resuscitation training.^14^ Despite low scores on the baseline confidence assessment, limited experience in leading a resuscitation and the absence of preassigned resuscitation leader role were more highly cited barriers to leading resuscitations than a lack of confidence. However, these barriers are interrelated. For example, a trainee with limited experience in resuscitation leadership may lack confidence in their ability to lead a successful resuscitation because of limited experience itself. If this trainee is not confident in their ability to take charge as the team leader, the lowest scoring item on the precourse confidence assessment, they may not organically obtain dedicated practice in this skill if their institution does not designate them as the resuscitation leader. If low confidence or self-efficacy prevents the learner from obtaining sufficient resuscitation experience, it may inhibit the development of competence.

Hospitals with resources for dedicated resuscitation teams are unlikely to revert to an ad hoc model given the association of designated resuscitation teams with improved performance.^1^ As such, alternative strategies are needed for developing resuscitation leadership skills in neurology learners. Senior neurology trainees who anticipate working in smaller or underresourced hospitals should be offered opportunities to rotate with resuscitation teams on an elective basis. Additional simulation training, through high-frequency, low-dose, or mastery learning protocols, which have been shown to improve cardiac arrest outcomes, would also lead to more resuscitation leadership experience.^14^ Either strategy could help mitigate the risk of knowledge decay after this brief simulation training and may be protective against sophomoric confidence, which could create a patient safety hazard. Although application of cardiac arrest leader training for development of neurology learner confidence and knowledge has not been previously evaluated, high-frequency, low-dose cardiac arrest management training has been described for NCC interdisciplinary teams.^11^ Interdisciplinary simulation training is effective in helping teams to understand and address the causes of team-based performance issues; however, acute situational training is more effective for promoting mastery of known skill sets.^21^ Psychological safety could be compromised in interdisciplinary simulation training for learners who lack confidence in resuscitation leadership, which may limit the beneficial outcomes (and potentially introduce harm) from the course. Although brief acute situational training (such as the current intervention) is unlikely to result in mastery of cardiac arrest leadership, the confidence gained may facilitate a psychologically safe transition into more frequent simulation training with the interdisciplinary team.

This shared simulation was effective in improving learner confidence and knowledge in cardiac arrest management, despite being designed for a different group of learners. These findings support the possible utility of developing a neurology simulation case library, as proposed by others and recently launched by *Neurology: Education*.^10,12^ Beyond neurology, clinician educators in other fields using shared formative simulations should have more confidence in the potential impact of these courses on desirable learner outcomes. Those interested in delivering new simulation-based educational interventions should consider first searching for existing interventions with shared learning objectives before investing the time to develop a new course de novo. Although this intervention highlights that shared simulations have the potential to improve learner knowledge and confidence, it does not guarantee that all shared simulations will have these effects. Future research should explore whether shared simulations can reliably improve learner performance and patient outcomes and whether these improvements are comparable with those of the learners for whom the simulation was originally designed.

The generalizability of these results is limited by the small sample size. Although enrollment occurred at 2 centers, most participants were from a single hospital system. Because the course was initially offered on an elective basis at both sites, there is a risk of self-selection bias. As above, only confidence and knowledge were measured so improvements in learner performance or patient outcomes cannot be inferred as a result of the intervention. The positive outcomes from this study, focusing on the management of a single disease, cannot necessarily be extrapolated to any disease state for which a simulation is shared. In addition, the lack of a control group makes it unclear whether the observed improvements in knowledge and confidence could have been achieved with a less time intensive educational intervention, such as a lecture.

Shared simulations improved learner confidence and knowledge in cardiac arrest resuscitation leadership and could offer similar benefits in other simulated scenarios. Low confidence, cited as a barrier to resuscitation leadership by over one-third of NCC fellows, was effectively improved through brief simulation training.

## Glossary

ACGME: Accreditation Council for Graduate Medical Education
ACLS: Advanced Cardiovascular Life Support
BWH: Brigham and Women's Hospital
CMS: Center for Medical Simulation
CPR: cardiopulmonary resuscitation
IHCA: in-hospital cardiac arrest
NCC: neurocritical care
PEA: pulseless electrical activity
ROSC: return of spontaneous circulation
VF: ventricular fibrillation
